# Towards a Bioactive Food Packaging: Poly(Lactic Acid) Surface Functionalized by Chitosan Coating Embedding Clove and Argan Oils

**DOI:** 10.3390/molecules26154500

**Published:** 2021-07-26

**Authors:** Elena Stoleru, Cornelia Vasile, Anamaria Irimia, Mihai Brebu

**Affiliations:** Physical Chemistry of Polymers Department, “Petru Poni” Institute of Macromolecular Chemistry, 41A Gr. Ghica Voda Alley, 700487 Iaşi, Romania; cvasile@icmpp.ro (C.V.); anamaria.sdrobis@icmpp.ro (A.I.)

**Keywords:** bioactive coating, surface functionalization, emulsion entrapment, UV protection, food spoilage

## Abstract

Here we introduce a new method aiming the immobilization of bioactive principles onto polymeric substrates, combining a surface activation and emulsion entrapment approach. Natural products with antimicrobial/antioxidant properties (essential oil from *Syzygium aromaticum*—clove and vegetal oil from *Argania spinosa* L—argan) were stabilized in emulsions with chitosan, a natural biodegradable polymer that has antimicrobial activity. The emulsions were laid on poly(lactic acid) (PLA), a synthetic biodegradable plastic from renewable resources, which was previously activated by plasma treatment. Bioactive materials were obtained, with low permeability for oxygen, high radical scavenging activity and strong inhibition of growth for *Listeria monocytogenes*, *Salmonella Typhimurium* and *Escherichia coli* bacteria. Clove oil was better dispersed in a more stable emulsion (no separation after six months) compared with argan oil. This leads to a compact and finely structured coating, with better overall properties. While both clove and argan oils are highly hydrophobic, the coatings showed increased hydrophilicity, especially for argan, due to preferential interactions with different functional groups in chitosan. The PLA films coated with oil-loaded chitosan showed promising results in retarding the food spoilage of meat, and especially cheese. Argan, and in particular, clove oil offered good UV protection, suitable for sterilization purposes. Therefore, using the emulsion stabilization of bioactive principles and immobilization onto plasma activated polymeric surfaces we obtained a bioactive material that combines the physical properties and the biodegradability of PLA with the antibacterial activity of chitosan and the antioxidant function of vegetal oils. This prevents microbial growth and food oxidation and could open new perspectives in the field of food packaging materials.

## 1. Introduction

Nowadays, the main concerns in the food industry are oriented towards two different directions: one is related to food safety, preservation and quality assurance and the other one includes the issue of packaging waste. The latter is determined by the fact that the food packaging sector is still dominated by fossil-based plastics (mainly non-degradable ones) with their related problems in disposal, recycling and incineration [1]. Therefore, biodegradable food-packaging is required to replace petroleum-based plastics. Among the biodegradable plastics, poly(lactic acid) (PLA) has gained recently a lot of attention in food packaging especially due to its biocompatibility, biodegradability under industrial composting conditions [2] and biological origin, being derived from various renewable agricultural resources [3]. Moreover, PLA is approved for use in food packaging, including direct contact applications, being classified as GRAS (Generally Recognized as Safe) by the U.S. Food and Drug Administration (FDA) [4], and also, more recently, authorized by the European Commission [5]. The main limitations of PLA in large scale production of food packaging are its high costs, poor properties as gas or liquid barrier [6] and high microbial adhesion [7]. Recently, the production cost of PLA has been lowered by using modern and emerging technologies, thus the applicability range in packaging was broadened [8,9]. The other drawbacks could be addressed by deposition of barrier and antimicrobial coatings onto PLA materials [10,11,12]. The addition of biological active compounds only at the surface of material allows tailoring favorable interactions at the interface, enhancing bioavailability while preserving the bulk properties. Moreover, this offers the advantage of reduced consumption of active substances compared with classical addition into material’s bulk [13,14].

The increasing demand for healthier and highly nutritional food, containing natural preservatives without compromising human and environmental safety, determines the necessity for development of new preservation methods and packaging technologies [15]. In daily life, food spoilage and deterioration are mainly caused by foodborne pathogens or other microorganisms, such as *Campylobacter*, *Salmonella*, *Yersinia enterocolitica*, *Escherichia coli* or *Listeria monocytogenes*, which grow mainly on food surfaces, or microorganisms growing in the pores of food contact materials [16].

A special attention is lately directed towards products derived from plants, which are rich in bioactive components, and can induce antimicrobial and antioxidant properties to the final materials [17,18,19,20]. From this group of bioactive substances, the essential and vegetal oils have gained a lot of interest in various domains, mainly based on their intrinsic properties such as antioxidant, antibacterial, antiviral, antifungal and insecticidal [21]. In the category of essential oils, clove (*Syzygium aromaticum*) stands out with a strong antioxidant activity, which is higher than that of some synthetic antioxidants such as butylated hydroxytoluene or butylated hydroxyanisole; this potential in particular is assigned to its high eugenol content [22]. Clove essential oil (CEO) also presents a broad antimicrobial activity towards various bacteria and fungi [23]. The virgin oil obtained from the argan tree (*Argania spinosa*) has a high content of linoleic and oleic acids and significant levels of polyphenols and tocopherols, which determines its antioxidant activity [24].

Melt processing is the usual industrial technique to obtain polymeric packages but it presents big technological challenges when intended for mass incorporation of bioactive compounds (e.g., essential or vegetal oils) into materials for packaging because of their high volatility or susceptibility to thermal degradation. Protection of bioactive principles can be achieved by choosing polymeric matrices with lower processing temperatures at which plant oils do not evaporate or degrade, or by inclusion into additional biopolymer matrices that are thermally resistant at the processing temperature of the base material. The encapsulation process is generally achieved by emulsification followed by solidification [25], and can prolong the bioactivity and diminish the impact on the organoleptic properties of foods [26].

Amongst natural biopolymers, the amino-polysaccharide chitosan shows promising features as the incorporation matrix of bioactive additives, such as plant-derived oils or extracts [27]. Chitosan has gained attention in this field due to its many unique properties such as good film-forming ability, edibility, biodegradability, biocompatibility, nontoxicity and antimicrobial activity against fungi, Gram-positive bacteria and Gram-negative bacteria [20,28,29,30]. Moreover, chitosan has the ability to interact through its functional groups with different compounds in plant oils, promoting their entrapment [31].

Several studies on incorporation of plant oils into chitosan-based matrices are reported in scientific literature [32,33,34,35] but, to our knowledge, only few works considered the immobilization of hybrid oil-loaded chitosan coatings onto the surface of packaging materials. In this view, we recently investigated the bioactivation of PLA surface by deposition of essential and vegetal oils encapsulated into chitosan matrix through co-axial electrospinning [36]. The electrospraying/electrospinning was the most versatile among various deposition methods; however, the wet coating method was the most efficient one in terms of homogeneity of surface and thickness of deposited layer [11,37,38].

We hypothesize that the complex composition of essential and vegetal oils, rich in bioactive compounds, will provide antioxidant potential and enhanced antimicrobial activity to chitosan-based coating, and prevent the development of antimicrobial resistance. Moreover, the immobilization of oil-loaded chitosan coatings onto PLA substrate will inhibit microbial adhesion and will improve the barrier properties, reflected by decreased gas permeability.

In this study we present a bioactive food packaging material based on poly(lactic acid) film tailored with chitosan coating that contains entrapped bioactive principles. The approach considers a synthetic biodegradable plastic substrate from renewable resources (PLA), a natural biodegradable polymer with antimicrobial activity (chitosan) and natural antimicrobial/antioxidant compounds (clove essential oil and argan vegetal oil).

## 2. Materials and Methods

### 2.1. Materials

As polymer substrate poly(lactic acid) (PLA), 2002D type, Mw = 217,000 g/mol, purchased from NatureWorks LLC, was used in a film shape (thickness of 0.3 mm ± 0.05 mm) obtained by melt processing and pressing using a Carver press at 175 °C. Chitosan (CHH) from crab shells, with high molecular weight (310,000–375,000 g/mol), deacetylation degree of 87.8%, as determined by ^1^H-NMR [11] and a dynamic viscosity of 1% solution in acetic acid higher than 400 mPa.s, and 1-ethyl-3-(3-dimethylaminopropyl) carbodiimide hydrochloride (EDC) (used as activator of hydroxyl groups) were acquired from Sigma-Aldrich, Steinheim, Germany. Four essential oils, namely *Syzygium aromaticum* (clove—CEO), *Thymus vulgaris* (thyme—TEO), *Rosmarinus officinalis* (rosemary—REO) and *Melaleuca alternifolia* (Ti Tree—TTO) were purchased from Fares, Orastie, Romania. Three varieties of cold pressed oils, namely *Vitis Vinifera* (grape seeds—GVO), *Argania spinosa* L. (argan—AVO) and *Prunus armeniaca* (apricot—APO) were purchased from Herbavit, Oradea, Romania. The oil with the highest antioxidant function in its class was chosen as best candidate to induce the required bioactivity for food packaging materials, together with the well-known antibacterial activity of chitosan. Oil-in-water micro-emulsions of vegetal oils with chitosan were obtained, further forming bioactive surface coatings for PLA film. Tween 80 (T80) from Sigma Aldrich, Germany, was used as emulsifier. Acetic acid and chloroform solvents were of analytical grade, purchased from Chemical Company, Iasi, Romania.

### 2.2. Formulation of Coating-Forming Emulsion

**Scheme 1:**
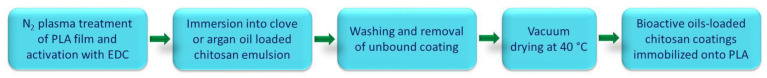
The flow chart showing the preparation of PLA films bio-functionalized with oil-containing coatings.

Chitosan solutions of 1.3% *wt*/*v* concentration were prepared by dissolving the required amount of the amino-polysaccharide in 2% *v*/*v* acetic acid and magnetic stirring at a speed of 250 rpm for 48 h. The obtained solutions were filtered before further use to remove the insoluble matter. Afterwards, volumes of 150 µL from each selected oil were added to 20 mL chitosan solution in presence of 0.018 g Tween 80 and homogenized into a glass beaker with an ultrasonic processor UP50H (Hielscher—Ultrasound Technology, Teltow, Germany) using a power of 50 W at 30 kHz frequency for 10 min. The tip of the ultrasonic probe was positioned at the center of the beaker, about 4 cm from the bottom. The processing conditions were selected following an optimization process. The process flow is graphically presented in Scheme 1.

### 2.3. PLA Activation and Functionalization

Due to the lack of easily reacting side chain groups and the hydrophobic nature of the PLA a prior functionalization of the polymer surface is necessary in order to facilitate the adhesion of bioactive coatings. Therefore, the PLA films were exposed to high-frequency plasma generated at low pressure (1.3 MHz, 40 Pa) inside a glass reactor using nitrogen gas atmosphere and 100 Watts discharge power. A detailed description of the plasma system is presented in a previously published article [12]. 

Immediately after plasma treatment the PLA films were immersed into EDC activated chitosan (the control sample, labeled as PLA/CHH) or oil-loaded chitosan solutions (coded as PLA/CHH/CEO or PLA/CHH/AVO) and left for 12 h at 4 °C. To facilitate the covalent bonding of chitosan coatings onto PLA surface, its hydroxyl groups were activated by adding EDC coupling agent dissolved in a small volume of phosphate buffer solution (pH 7.4), keeping the CHH/EDC ratio at 30/1 (*g*/*g*). The coated PLA films with chitosan or oil-loaded chitosan formulations were excessively rinsed, first with 1% aqueous acetic acid solution, next with water, to remove the unbound compounds, and finally vacuum dried at 40 °C. The samples thus prepared were kept in a desiccator until further analyses were performed.

### 2.4. Characterization

#### 2.4.1. FTIR Spectroscopy

A VERTEX 70 spectrometer (Bruker Optics, Ettlingen, Germany) was used to record the Fourier-transform infrared spectra (FTIR) in the 600–4000 cm^−1^ domain, at a spectral resolution of 4 cm^−1^ by co-adding 64 scans. Attenuated Total Reflectance (ATR) sampling technique was applied, the instrument being equipped with a ZnSe crystal at 45° angle of incidence. Prior to each measurement a background spectrum was recorded. The processing of spectra was achieved using OPUS software.

#### 2.4.2. Scanning Electron Microscopy

The morphology of polymeric surfaces was evaluated by scanning electron microscopy (SEM) using an electron microscope VEGA II TESCAN (Brno, Czech Republic, without any treatment.

#### 2.4.3. Contact Angle

The wettability of surfaces was determined by static contact angle measurements performed on a CAM-200 goniometer from KSV-Finland. The water contact angle (WCA) was determined by the sessile drop method, at room temperature and controlled humidity, within 2 s after placing 1 μL drops of liquid on sample’s surface. All reported contact angle values are average results from at least 10 measurements on different places on the surface. 

#### 2.4.4. Gas Permeability

Oxygen permeability was determined with a PERMETM OX2/231 Permeability Tester from Labthink Instruments CO., LTD (Jinan, China), at 23 °C and ~50% RH. Oxygen transits through the film and is delivered to the sensor by nitrogen carrier gas. The oxygen flow rate was fixed at 20 mL/min, while that of nitrogen was 10 mL/min.

#### 2.4.5. Antioxidant Activity

The antioxidant activity was determined by testing the radical scavenging potential of the vegetal oils and of the coated PLA films against 2,2′-azino-bis 3-ethylbenzthiazoline-6-sulfonic acid (ABTS) and 2,2-diphenyl-1-picrylhydrazyl (DPPH) radicals, according to protocols adapted for liquid and solid substances.

(a) Evaluation of vegetal oils.

Briefly, ABTS•^+^ cation radical is generated by mixing stock aqueous solutions of ABTS (7 mM) with a strong oxidizing agent, namely potassium persulfate (14.7 mM), in a 1:1 volume ratio, followed by storage in the dark at room temperature for 16 h. The blue -green ABTS•^+^ chromophore has strong absorption at 750 nm and in the presence of antioxidant agents can be neutralized, thereby resulting in a decrease of absorbance. The ABTS•^+^ solution was diluted with ethyl alcohol (purity of 99.3%) to an absorbance of 0.7 at 750 nm [39,40].

DPPH is a stable free radical with an intense violet color that under the action of proton donating compounds changes to light yellow. The extent of discoloration of dark violet color is quantified as the decrease in absorbance at 517 nm.

In order to calculate the IC_50_ values (the concentration at which the inhibition of ABTS•^+^ or DPPH free radical activity is at 50%) of selected plant oils, individual solutions were prepared at concentrations varying from 0.156 to 20 mg/mL for essential oils in methyl alcohol and from 1.25 to 40 mg/mL for cold pressed oils in trichloromethane [41]. The IC_50_ was calculated using linear regression analysis. All tests were conducted in duplicate. 

(b) Evaluation of PLA surface modified films.

PLA-based films with or without oil-loaded chitosan coating (squared samples of 10 cm^2^, ≈0.4 g) were submerged into 6.0 mL of 0.05 mM DPPH. The solutions did not need filtering since no dissolution occurred in the solvent used. A particular volume of the DPPH solution where the polymeric films were soaked was withdrawn from the testing vessel and afterwards UV-Vis spectra were recorded. The absorbance was measured at 517 nm after 30 min of incubation in dark [42].

The radical scavenging activity (RSA %) was calculated using Equation (1):

RSA (%) = (A_control_ − A_sample_)/A_control_ × 100 (1)

where A_sample_ is the absorbance of radical solutions (DPPH) incubated with the PLA-based samples and A_control_ is the absorbance of control (DPPH solution without the polymeric sample). 

#### 2.4.6. Evaluation of Microbiological Properties

(a) In vitro antimicrobial activity by colony count method.

The antibacterial effect of PLA films was assessed using the colony counting method (CCM) based on the standard procedures, namely SR ISO 16649-2/2007 for *E. coli*, SR EN ISO 11290-1:2000/A1:2005 for *Listeria monocytogenes*, and SR EN ISO 6579/2003/AC/2004/AC/2006, amendment 1:2007 for *Salmonella* bacteria [11]. The counting method involves the color reaction with 5-bromo-4-chloro-3-indolyl-β-D-glucuronide for *E. coli*, β-haemolysis test for *Listeria monocytogenes* and the specific plate count Xylose Lysine Deoxycholate agar (XLD agar) for *Salmonella*.

All bacteria used in the assay were from American Type Culture Collection (Rockville, MD, USA). Three types of bacteria, specifically *Escherichia coli* (ATCC 25922) and *Salmonella typhimurium* (ATCC 14028), which are Gram-negative, and *Listeria monocytogenes* (ATCC 7644), which is a Gram-positive bacterium, were tested. These bacteria are considered common food pathogens that may contaminate both animal and non-animal foodstuffs. Shortly, the bacterial strains have been reconstituted according to specific standards, namely SR EN ISO 11133:2014, ILAC G9/2005, SR EN ISO 7218-A1/2014. The polymeric films were sterilized in autoclave (for 10 min at 110 °C), and then 0.1 mL (10^2^–10^3^ CFU) of each microbial suspension was placed on top of the sample and incubated at 37 °C for 24 h, after which the bacterial colonies were counted. A log reduction of 10 log CFU/g of 0.052 is applied for each experiment, which was performed in triplicate. While autoclaving might have an adverse impact on free bioactive compounds, we expected the effect on the fixed/immobilized ones into biopolymer coating to be neglectable.

(b) Microbiological analysis of raw beef meat and white cheese.

Unmodified or surface modified PLA samples by oil-loaded CHH coatings were shaped in square forms with the same surface area and UV sterilized in Petri dishes. Tests were carried out on sliced beef meat kindly provided by a local slaughter house, delivered no later than 4 h after slaughter, or on sliced curd cheese from one dairy plant selected at random. The organoleptic parameters of the tested beef meat were red, moist, odorless and elastic appearances. The initial pH of the meat was 5.9, had a 10 mg/100 g content of easily hydrolysable nitrogen, manifested negative Kreiss reaction and did not react with hydrogen sulfide. The cheese used for testing was freshly obtained from curdled cow’s milk by adding rennet as coagulant. The fresh control cheese sample was characterized by total viable counts less than 10 CFU/g and a pH of 5.0. Beef meat or cheese pieces were aseptically cut to 1 cm^3^ dimension using a specific slicer, placed onto polymeric samples, sealed and refrigerated at 7 °C. After 24 and 48 h of storage, the beef meat and cheese were evaluated for total viable counts (ISO 4833:2003 and SR EN ISO 7218/2014; Plate Count Agar at 30 + 1 °C for 72 h in aerobic conditions). The total plate count is a good indicator for the overall bacterial load. Critical hygienic conditions of fresh meat are reached when the total number of bacteria is found between 3.5 and 5 log CFU/cm^2^ [43]. Most often, the microbiological spoilage of white cheese during its storage is caused by yeast and molds. The white cheese examined was characterized by a low contamination (less than 10 CFU/g) with coli group bacilli, which are indicators of the hygienic quality.

#### 2.4.7. Statistical Analysis

Tests were performed in triplicates and results were expressed as average values and standard deviation. Analysis of variance (ANOVA), calculated by Matlab software (Matlab R2017B, MathWorks, Portola Valley, CA, USA was applied to determine the significant differences among samples, comparing the mean values with a significance of 0.05.

## 3. Results and Discussions

Four essential oils and three cold-pressed oils were evaluated previously [44] for their antioxidant activity as presented in Table 1. The results showed that the DPPH and ABTS free radicals were scavenged by all plant oils in a concentration dependent manner. It was found that clove and argan had lowest IC50 values among tested essential and vegetal oils, respectively.

Clove essential oil selection for this study laid also on its general recognized efficient antioxidant, anti-inflammatory, antibacterial, antifungal and antiviral properties [45] and on our previous findings on its intense antioxidant and antimicrobial activity [41]. Among cold pressed vegetal oils, argan was selected in addition to IC50 values for its good stability [46], high content (above 600 mg/kg) of tocopherols powerful antioxidants [47] and largely accepted general benefits.

### 3.1. Preliminary Evaluation of the Emulsions

Chitosan contains both hydrophilic D-glucosamine structural units formed by deacetylation of chitin and hydrophobic N-acetylated residues, offering the ability to form emulsion systems; therefore, it is used as emulsifier in the food industry, to uniformly stabilize oil droplets [48]. Ultrasonication for 10 min led to white emulsions, slightly turbid for clove essential oil but opaque for argan cold press oil. A notable phase separation was observed for the AVO-based system at about 2 months from the preparation, while no visible change was noticed for the CEO-based system even after 6 months. Argan vegetal oil consists of over 99 wt% acyl glycerides (mainly triglicerides), in addition to which valuable compounds such as tocopherols are presented in high amounts [49]. The highly hydrophobic nature of argan oil does not allow strong interactions with the hydrophilic functional groups of chitosan, explaining the observed behavior in time of the microemulsion. AVO-based emulsion presented a low zeta potential of ~19 mV and large droplets with average diameter of ~236 nm (Table 1), indicating an emulsion with limited stability and possible tendency towards droplets coalescence. On the other hand, clove essential oil has a relatively simple composition, consisting mainly of eugenol/eugenol acetate (85.7%/7.9%), β-/α-caryophyllene (4.5%), and very small quantities (below 2%) of few other sesquiterpenes, as previously determined by gas chromatography analysis [41]. Strong interactions occur between the hydrophilic compounds in CEO and chitosan, leading to good dispersion and stabilization of oil droplets in emulsion. This was sustained by the DLS analysis that indicated a unimodal, narrow distribution of droplets with average diameter of 12.1 nm and a high zeta potential of 34.3 mV.

### 3.2. Coatings Characterization

#### 3.2.1. Morphology

**Figure 1:**
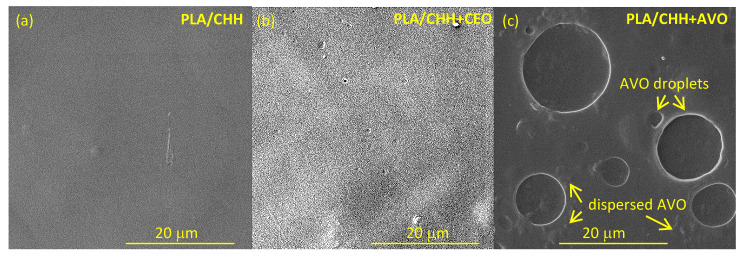
SEM micrographs of PLA samples surface modified with plant oil-loaded chitosan coatings: (**a**) PLA/CHH, (**b**) PLA/CHH-CEO, and (**c**) PLA/CHH+AVO.

Scanning electron microscopy examination of the PLA film modified with only chitosan reveals a smooth and continuous structured surface (Figure 1a), which is different to the PLA surface without any organized feature distinguished at SEM level of observation. Adding clove oil did not significantly change the surface morphology (Figure 1b), with homogeneous and uniformly distributed micro-domains only slightly larger in size compared with the chitosan coating but certainly below the micrometric scale that can be distinguished by SEM. This indicates a very fine dispersion and fixation of clove essential oil into chitosan matrix even after the evaporation of the solvent. Inspection of PLA film with argan oil-loaded chitosan showed several domains of non-uniformly dispersed oil in the chitosan matrix, and the presence of large circular domains, attributed to argan droplets of various sizes (between 2.1 and 7.2 µm in diameter, as measured on ~50 droplets from three different regions of the sample) into the coating layer (Figure 1c). Similar surface microstructure, namely discontinuities associated with the formation of two phases, was found for quinoa protein-chitosan-sunflower oil films by Valenzuela et al. [50]. It appeared that AVO dispersion in chitosan emulsion is destabilized during coating formation by solvent evaporation. In addition, the plasma treatment increased the hydrophilic character of the PLA, which induced the tendency of hydrophobic AVO to exudate far from the substrate–coating interface.

#### 3.2.2. Chemical Structure of Oil-Loaded Chitosan Coatings

**Figure 2:**
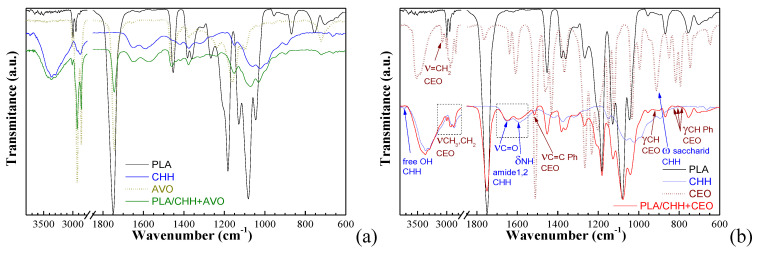
FTIR spectra of plasma treated PLA surface modified with chitosan coatings loaded with argan oil (**a**) and clove oil (**b**).

Characterization by FTIR spectroscopy was conducted to determine the interactions between the functional groups of chitosan and plant oils. The corresponding spectra are shown in Figure 2.

The FTIR spectrum of a chitosan film obtained in the presence of Tween 80 displays a broad band between 3600 cm^−1^ and 3200 cm^−1^, with two absorption maxima located at 3353 cm^−1^ and 3295 cm^−1^, assigned to O-H and N-H stretching (Figure 2—CHH). The vibration bands found at 2921 cm^−1^ and 2867 cm^−1^ are attributed to symmetric CH_3_ stretching and asymmetric CH_2_ stretching. The bands at 1650 cm^−1^ and 1587 cm^−1^ are characteristic bands of chitosan designating the stretching vibrations of the carbonyl groups (νC=O Amide I) and the bending vibration of the amino groups at the C_2_ position of glucosamine (ν NH_2_ Amide II) [51,52]. The bands occurring at 1375 cm^−1^ and at 1313 cm^−1^ are ascribed to overlap vibrations of CH bending and symmetric CH_3_ deformation, and to Amide III band and CH_2_ wagging, respectively. The bands characteristics to the saccharide structure of chitosan are found at 1150 cm^−1^ and 1019 cm^−1^ and are mainly assigned to asymmetric C–O–C bridge stretching and asymmetric in-phase ring stretching [53]. 

For argan oil (CAO) the IR spectrum (Figure 2a) reveals that in the 3100–2800 cm^−1^ range the major absorption bands are located around the frequencies of 3007, 2923, 2853 cm^−1^. The band at 3007 cm^−1^ is specific to the methyl-oleate, while the other absorptions bands are characteristic to the symmetrical and asymmetrical vibrations ν_C-H_ of the CH_2_ and CH_3_ aliphatic groups from the long alkyl chains, which are predominant in vegetal oils. The absorption at 1743 cm^−1^ is assigned to νC=O from RC=OOR moieties of triglycerides. The spectral band near 1654 cm^−1^ corresponds to the double C=C bonds and may be correlated with the content of polyunsaturated fatty acids in the oil. At 1461 cm^−1^ a band associated to the vibrations of deformation δ CH is noticed. Another two bands are observed at 1376 and 1237 cm^−1^; the first band corresponds to the deformation vibration in the phase of CH_2_ group, while the second band corresponds to the deformation vibration in the plan of the group =CH, from the double unconjugated *cis*-bonds [54].

Significant differences are observed in FTIR spectra of the PLA coated with AVO-loaded chitosan. In the case of PLA substrate surface modified with chitosan/argan oil mixture some characteristic bands of PLA [39] and of chitosan overlaps. Some characteristic band for argan oil, namely at 1462 and 1377 cm^−1^, attributed to vibrations of deformation δCH, and deformation vibration in the phase of methylene group could be clearly evidenced, indicating the successful fixation into the coating. 

The vibration bands identified in the FTIR spectrum of clove essential oil are consistent with the presence of the compounds identified by gas chromatography and are mainly associated with the specific vibrations of the following functional groups: phenolic hydroxyl stretch (at 3520, 3450 cm^−1^), carbon-hydrogen stretching from aromatic moiety (2938 cm^−1^), carbonyl νC=O in eugenol acetate (1763 cm^−1^), aromatic carbon–carbon stretching (1510 cm^−1^), carbon-oxygen stretch from ether group (1264 cm^−1^) and alkene =C-H bending (1034 cm^−1^) [55,56]. 

The FTIR spectrum of the clove oil loaded chitosan coatings show the successful embedding of CEO within chitosan matrix (Figure 2b). In particular, the νC=C at 1516 cm^−1^ and the γCH at 850 cm^−1^, 817 cm^−1^, 793 cm^−1^ vibration bands of the aromatic ring, and the νCH and γCH of terminal =CH_2_ group in eugenol at 3073 cm^−1^ and 913 cm^−1^ were observed as clear peaks or as shoulders in the FTIR spectrum of the coating in the range where no absorption bands of PLA substrate or chitosan matrix was observed. In addition, the bands in the 3100–2800 cm^−1^ range showed the overlapping of νCH vibrations in CH_3_, CH_2_ and =CH_2_ groups coming from PLA substrate, chitosan coating and clove essential oil loading.

The band at 892 cm^−1^ in the FTIR spectrum of chitosan film, corresponding to the wagging vibration of the saccharide structure [57,58,59,60], moved to 898 cm^−1^ in the oil-loaded coating. This shift at higher wavenumbers indicates a partial relaxation of chitosan macromolecular chains through interactions with low molecular weight compounds in CEO. Change in shape and intensities were observed for the oil-loaded chitosan coating in the 1700 cm^−1^–1500 cm^−1^ range of the spectrum, where the large band with a peak value around 1593 cm^−1^, standing for δNH and νC-N amide II in acetylated glucopyranose units and δNH_2_ amine in deacetylated ones [61], decreased in intensity becoming smaller than the band at 1651 cm^−1^ (νC=O amide I), compared with the same bands in chitosan film. An explanation could be the conversion of primary amine groups involved in H-bonding between the macromolecular chains in chitosan film into secondary amine groups, with weaker absorption at smaller wavenumbers, by interactions with components of clove essential oil in the coating. In addition, two small peaks were observed at 3735 cm^−1^ and 3708 cm^−1^ in the chitosan film. These are associated with –OH stretching vibration of the free hydroxyl groups from C6 and C3 position respectively in the amino polysaccharide structural unit. These peaks were not observed in the oil-loaded coating, suggesting the involvement of the -OH groups in intermolecular hydrogen bonding with PLA substrate or the clove oil loading. 

#### 3.2.3. Stability to UV Exposure

The PLA films, uncoated or coated with oil-loaded chitosan, were exposed for 30 min to UV radiation of 254 nm, which is proven to destroy bacteria and viruses. This was chosen to simulate the sterilization stage and to evaluate the stability of the obtained materials in conditions of sterilization that are widely applied in food packaging industry and for disinfection of food-contact surfaces. The optical properties before and after exposure were discussed based on the (CIE) L*a*b* color space parameters, that describes lightness by L* and the relative position to green–red and blue-yellow by a* and b*, respectively. The global color change is expressed by the ΔE parameter, calculated using the equation ΔE = [(ΔL*)^2^ + (Δa*)^2^ + (Δb*)^2^]^1/2^.

**Figure 3:**
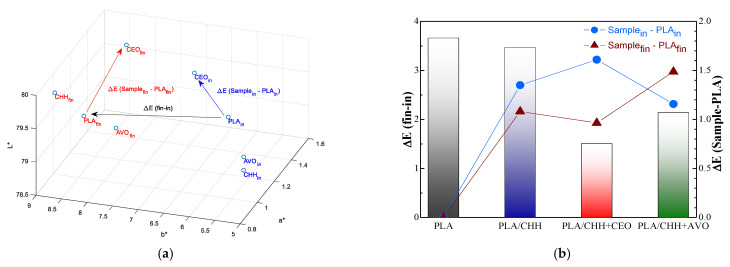
The L*, a*, b* color parameters (**a**) and the color change (**b**) of the coated PLA films before (in) and after (fin) UV irradiation for 30 min at 254 nm.

Figure 3 showed that chitosan-based coatings did not significantly affect the optical aspect of the PLA film. The color change induced by the coating (ΔE Sample_in_ − PLA_in_ in Figure 3b) was in the ~1.0–1.6 range. The highest difference was observed for clove oil-loaded chitosan, which shifted the b* parameter from ~5.4 to 6.9 (Figure 3a) due to the yellowish color of the pristine oil. The exposure at UV radiation for 30 min caused a slight yellowing effect, the b* value increasing from below 7 to above 8 (Figure 3a). The effect of radiation on the components of the coating could be observed from the ΔE Sample_fin_ − PLA_fin_ values in Figure 3b. The chitosan coating showed a color change of ~1.1 compared with uncoated PLA, due to the susceptibility of the polysaccharide chain to UV degradation. The color change was much higher (~1.5) for the AVO-loaded chitosan coating, due to the UV sensible double bonds in unsaturated fatty acids, which are in high amounts (up to 80%) in argan oils [62]. On the other hand, it seemed that the phenolic functional groups in clove essential oil could diminish the UV effect; the color change compared with the uncoated PLA was of 0.96, smaller that for the chitosan coating without embedded oil. The overall change (ΔE (fin-in) in Figure 3b) was small, below 3.7. Plant oils showed a UV protection effect, the ΔE decreasing to 2.14 for AVO and to 1.51 for CEO. Similar findings regarding the UV protection were reported also by incorporation of orange essential oil into carrageenan/trehalose-based films [63].

#### 3.2.4. Gas Barrier Properties

Is well known that for materials intended to be used in food packaging good barrier properties are required. A barrier polymeric material is most commonly used in the packaging industry to stop the passage of small molecules of gases, moisture or flavors. The barrier properties of a polymeric film are important because it is necessary to preserve the quality of food and improve its shelf-life [64]. Generally, gas permeability depends on the level of arrangement of the polymer chains, the film microstructure (holes or discontinuities in the polymer structure), thickness and the solubility of gases in the material [65].

**Figure 4:**
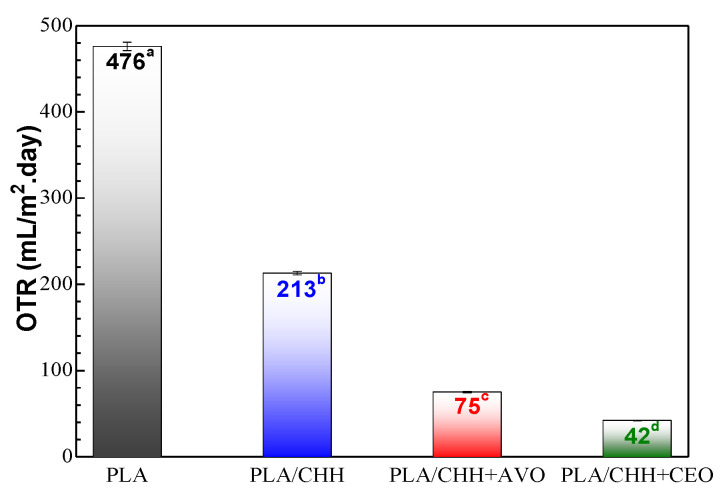
Oxygen transmission rate (OTR) of PLA-based films and surface modified with oil-loaded chitosan coatings. The data are expressed as mean ± standard deviations from triplicate determinations. ^a–d^ Mean values with different letters indicate significant differences at *p* < 0.05.

Figure 4 introduces the oxygen transmission rate (OTR) values for native and surface modified PLA-based samples. Gas permeability tests have shown that the oil-loaded chitosan-coated films were significantly less oxygen permeable than PLA substrate, the lowest OTR value is noticed for the PLA/CHH + CEO sample. The rather high oxygen permeability of PLA was sharply decreased with about 55% by coating with chitosan. This could be explained by the dense structuration at micro-scale, as noticed in Figure 1a. Since the addition of clove essential oil has strong interactions with the chitosan and maintains the compactness of the structure (Figure 1b) the oxygen permeability is further decreased, from 213 to 42 mL/m^2^.day. Coalescence of argan oil in droplets (as seen in Figure 1c) explains higher oxygen permeability of the coating compared with clove oil containing system.

#### 3.2.5. Wettability Evaluation

**Figure 5:**
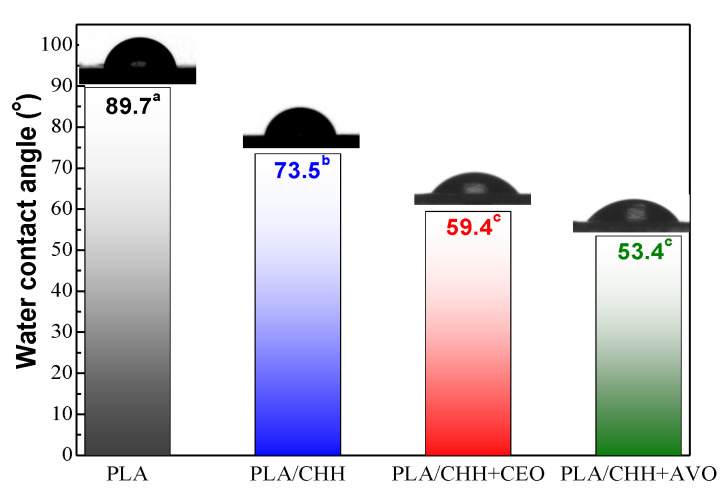
Water contact angle of PLA film with oil-loaded chitosan coatings. The data are expressed as mean ± standard deviations from triplicate determinations. ^a–c^ Mean values with different letters indicate significant differences at *p* < 0.05.

For all the obtained samples water contact angle (Figure 5) was measured to evaluate the wettability of surface modified samples. Water contact angles are also illustrated by the photographs of water droplets deposited on different surfaces. The PLA substrate is close to the hydrophobicity boundary, generally considered at 90°, and by chitosan coating, the surface becomes slightly hydrophilic, with a measured contact angle of 73.5°. Plasma treatment creates reactive sites onto PLA surface that are involved in chemical bonding with chitosan in coatings. By oil-loading the coatings, the hydrophilicity of the PLA-based samples further increased, behavior that at first glance seems intriguing, taking into account the fact that the oils are hydrophobic by their nature. The hydroxyl and amino moieties are involved in H- type intra-molecular bonding, conferring a moderate hydrophilicity to chitosan. These are affected by the presence of plant oils, especially by CEO, which induce strong inter-molecular interactions, mainly with the amino groups, as observed by FTIR. These liberate the -OH groups that can associate themselves, improving the hydrophilicity of the surface. As a result, the water contact angle decreased from 73.5° to 59.4°. On the other hand, the triglyceride -OC=O groups in argan oil could preferentially interact through H- bonding with the hydroxyl groups of chitosan, making more available the amino groups, with increased hydrophilicity. Therefore, the water contact angle of the AVO-containing coating had the lowest value, of 53.4°. The results obtained are consistent with other similar systems reported in the scientific literature. Souza et al. [66] found that ginger essential oil incorporation into chitosan-based films had increased surface hydrophilicity and this behavior was attributed to the presence of phenolic compounds in the oil, which promoted the interaction with water. Wen et al. have evidenced that addition of cinnamon essential oil embedded into β-cyclodextrin have dropped the water contact angle of poly(lactic acid) nanofilm [67]. The rise in hydrophilicity of AVO-loaded chitosan coating may be due to the amphiphilic property of the amino-polysaccharide which stabilizes the oil by inverse micelles formation. In this way the surface of coatings is enriched with the hydrophilic regions of micelles and the polar groups being more available for interaction with water. Equivalent findings were evidenced also by Rullier-Birat et al. [68] when incorporating beeswax into emulsified chitosan-based coatings. Slightly higher value of the contact angle in the case CEO-loaded coating compared with AVO-loaded one can be attributed to the stronger interactions that occurred between the main compound of clove oil (eugenol) and chitosan. These strong interactions let to a decrease in the number of the chitosan hydrophilic functional groups available at the upper surface of the coating (solid-air interface).

#### 3.2.6. Radical Scavenging Potential

**Figure 6:**
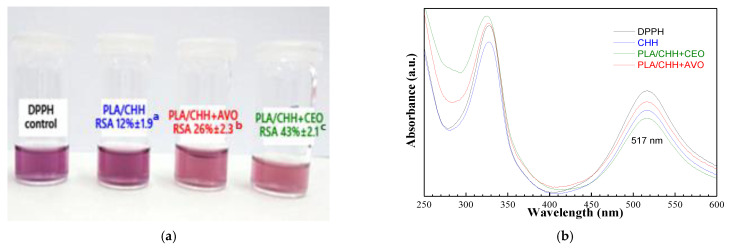
Photographs of DPPH solutions after 30 min dark incubation with/without polymeric samples (**a**) and UV-Vis spectra of DPPH scavenging by PLA-based samples unmodified and modified with oil-loaded coatings (**b**). ^a–c^ Mean values with different letters indicate significant differences at *p* < 0.05.

The assessment of the antioxidant potential of the samples was achieved by determining the inhibition capacity of free DPPH radicals. DPPH absorbs strongly at 517 nm, its solution having a dark purple color (Figure 6a). The potential for free radical scavenger was determined by measuring the reduction of absorption after 30 min from the addition of the samples to the DPPH solution. As expected, PLA film had no scavenging activity towards the DPPH radical; however, the chitosan coating imparts a low activity, of 12%. Incorporating plant oils into chitosan coating enhanced the radical scavenging for DPPH, indicating that the bioactive compounds preserved their effectiveness along the obtaining process. The clove essential oil strongly increased the RSA of the chitosan coating to 43%, due to the presence of high amounts of eugenol. The bioactive tocopherols are minor compounds in argan oil; therefore, the RSA of AVO-loaded chitosan coating had an intermediary value of 26% (Figure 6b).

#### 3.2.7. Antimicrobial Results

Pathogenic microorganisms as *Listeria monocytogenes*, *Salmonella Typhimurium* or *Escherichia coli* (*E. coli*) O157:H7 are of concern under the point of view of food safety. Those microorganisms are present in a broad range of foodstuffs contributing every year to large transmission of food borne illnesses [69].

**Figure 7:**
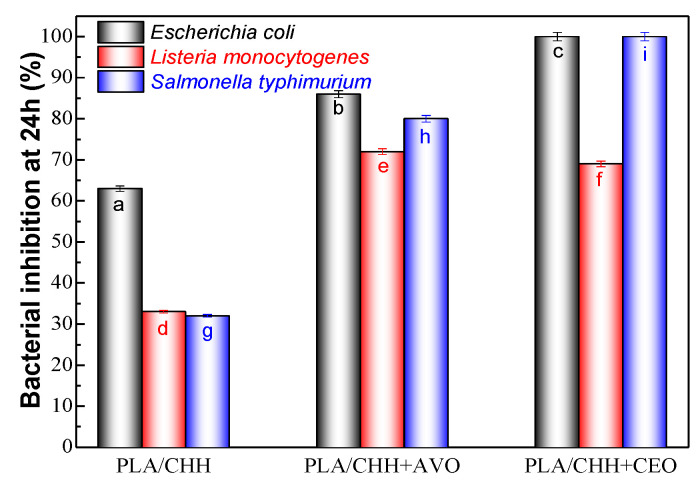
Variation of bacteria percentage inhibition determined by the CHH and oil-loaded CHH coatings. The data are expressed as mean ± standard deviations from triplicate determinations. ^a–i^ Mean values with different letters indicate significant differences at *p* < 0.05.

The results in Figure 7 showed that besides the intended antioxidant effect, the plant oils also significantly contribute to the antibacterial activity of the coatings. It appeared that the clove oil-loaded chitosan coatings manifested a more pronounced overall bacterial inhibitory effect—Figure 7, except for the case of *Listeria monocytogenes* species, where argan-loaded chitosan coating was slightly more effective. For the clove oil-loaded chitosan-coated sample, the following microbial susceptibility order was turned out: *S. Typhimurium* > *E. coli* > *L. monocytogenes*, while for argan oil-loaded chitosan coating the susceptibility order was: *E. coli* > *S. Typhimurium > L. monocytogenes.* A synergistic antibacterial effect was noticed by the incorporation of both plant oils into chitosan-based coatings, similar results being reported in previously published papers [36]. It is important to notice that, even if the samples were sterilized by autoclaving, they manifested the intended bacterial inhibition properties, thus a possible negative effect of the sterilization could be neglected.

#### 3.2.8. Microbiological Analysis Results

**Figure 8:**
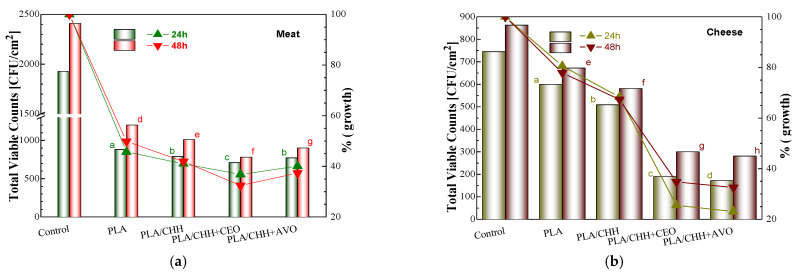
Variation in time of Total Viable Counts for beef meat (**a**) and white cheese (**b**) stored in contact with poly(lactic acid) unmodified and modified with oil-loaded chitosan coatings. The data are expressed as mean ± standard deviations from triplicate determinations. ^a–h^ Mean values with different letters indicate significant differences at *p* < 0.05.

Microbiological tests were performed to evaluate the potential applicability of the obtained PLA films coated with oil-loaded chitosan as food contact packaging materials. Two types of food, meat and cheese, were tested in terms of dynamics of spoilage-related microorganisms. Microbial growth (Total Viable Counts—TVC) was monitored for fresh beef meat and white cheese stored in refrigerator in contact with PLA coated with oil-loaded chitosan. Since microbial growth is strongly dependent on food type, data were also expressed as percentage growth considering control samples as reference. These data were represented on the right axes in Figure 8.

Oil-loading into chitosan coatings leads to a significant decrease of Total Viable Counts when compared with the PLA substrate surface modified only with chitosan (PLA/CHH). Differences can be observed between the activity on meat and on cheese, as well as on microbial growth in time. A decreased TVC was noticed when the bottom side of food was in contact with the PLA film instead of being placed directly on the glass Petri dish. This does not imply an antimicrobial action of the PLA itself, but rather an inert effect, due to the hydrophobic character compared with the glass surface. Interestingly, this inert effect was higher for meat (only 45–50% microbial growth) compared with cheese (~80% microbial growth), both at 24 h and at 48 h. Chitosan decreased the TVC; however, this effect was rather lower than expected, considering its well-known antimicrobial activity revealed by various performed researches on food products [27]. An explanation might be the chemical immobilization of chitosan onto plasma-activated PLA substrate [11]. Incorporation of plant oils into chitosan coating further improved the microbial inhibition. The effect was much stronger in the case of cheese, with a sharp decrease of growth percentage from about 68% to below 25% after 24 h and below 35% after 48 h. This could be explained by the different pH of the two types of food, namely ~5 for cheese and ~6 for meat, the release of active principles from the coating being favored below the pKa of chitosan that is at 5.5, when the protonation of amino groups leads to the relaxation of the structure. Argan vegetal oil had slightly more effect than clove essential oil on cheese, while the opposite was observed on meat. The qualitative sensorial analysis was performed after 48 h and has revealed no noticeable migration of the bioactive oils to the food. In conclusion, both clove and argan oils loaded into chitosan coatings proved to be valuable antimicrobial agents for delaying the spoilage of the tested food.

## 4. Conclusions

Bioactive clove and argan oil-loaded chitosan coatings onto plasma-treated PLA films were obtained by emulsion entrapment approach. The good homogeneity of the coatings and the encapsulation of the oil were ensured by the presence of the Tween 80 emulsifier and ultrasonication. In the case of clove oil-loaded chitosan, a homogenous and uniform distribution of the oil droplets into the chitosan coating was noticed, with an average size much lower than in the case of argan oil. The FTIR spectroscopy demonstrated both the clove and argan oil embedding into chitosan coating and successful immobilization onto plasma functionalized PLA film. Strong interactions occurred between the phenolic compounds in clove oil and chitosan. Coating deposition onto PLA did not significantly change the color compared with the pristine polymeric film. Plant oils embedded into chitosan coatings determined a UV protection effect, more noticeably manifested by clove oil, suggesting that these materials are stable at UV sterilization. Oxygen permeability of PLA film is lowered by coating with oil-loaded chitosan, with the sharpest decrease being noticed in the case of clove oil loaded system, mainly due to its compact structure. The hydrophilicity of the PLA is enhanced by oil incorporation into coating, the lowest water contact angle value being observed for argan. This property is related to the preferential interaction of the triglyceride ester groups in argan oil with the hydroxyl groups of chitosan, increasing the availability of the hydrophilic amino groups at the upper surface. The oil-loaded coatings thus obtained conferred antioxidant and antimicrobial activity to the otherwise inert PLA and proved to successfully delay the meat and cheese spoilage. Incorporation of bioactive oils in chitosan coatings is a more convenient method than direct oil fixation onto the PLA surface, mainly because it retains antimicrobial/antioxidant activities for a longer time. The main advantage of this approach relies in the fact that while the coated side of the polymeric material inhibits the bacterial development the uncoated PLA side assures its further biodegradability. The research performed in this study may represent a sustainable approach in developing a bioactive food packaging material.

## Figures and Tables

**Table 1:** The radical scavenging ability (IC50) of the selected essential and vegetal oils and characteristics of CEO and AVO chitosan emulsions.

Essential/Vegetal Oils	DPPH IC_50_, mg/mL	ABTS IC_50_, mg/mL
Clove (CEO)	0.017 ± 0.005 ^a^	0.008 ± 0.002 ^a^
Thyme (TEO)	0.056 ± 0.007 ^a^	0.056 ± 0.009 ^b^
Rosemary (REO)	0.322 ± 0.081 ^b^	4.442 ± 0.012 ^c^
Ti Tree (TTO)	0.853 ± 0.121 ^c^	3.793 ± 0.023 ^d^
Argan (AVO)	10.416 ± 0.963 ^d^	5.279 ± 0.052 ^e^
Apricot (APO)	14.722 ± 0.825 ^e^	6.961 ± 0.127 ^f^
Grape Seeds (GVO)	16.784 ± 1.223 ^f^	7.129 ± 0.861 ^f^
**Emulsion**	**ζ-Potential (mV)**	**Droplet Size (nm)**
CHH + CEO	34.32 ± 1.2 ^a^	12.1 ± 1.6 ^a^
CHH + AVO	18.64 ± 2.3 ^b^	236.2 ± 16.4 ^b^

Mean ± standard deviations from triplicate determinations. ^a–f^ Mean values with different letters in the same column indicate significant differences at *p* < 0.05.

## Data Availability

Not applicable.

## Sample Availability

Not available.
